# Parental Leave Perceptions Among Emergency Medicine Residents and Faculty in India: A Multicenter Observational Study

**DOI:** 10.7759/cureus.113151

**Published:** 2026-07-22

**Authors:** Saumya K Shanmugathai, Narendra Jena, Joseph D Pauly, Nancy M Saral, Janice Blanchard

**Affiliations:** 1 Emergency Medicine, Meenakshi Mission Hospital and Research Centre, Madurai, IND; 2 Emergency Medicine, George Washington University School of Medicine and Health Sciences, Washington, D.C., USA

**Keywords:** emergency medicine residents, faculty perceptions, gender bias, india, maternity leave, parental leave, paternity leave, work-life balance

## Abstract

Background: Many residents become parents during residency, and the adequacy of parental leave is closely related to resident well-being. To the best of our knowledge, limited research has evaluated Emergency Medicine (EM) residents and faculty members' perceptions regarding parental leave during residency training in India. This study was conducted to analyze perceptions of Master of Emergency Medicine (MEM) residents regarding parental leave, assess faculty perceptions of parental leave and its impact on residency training, and evaluate respondents’ knowledge of existing maternity and paternity leave policies.

Methods: A multicenter prospective observational study was conducted from September 2022 to January 2023 among EM residents and faculty members working in tertiary care hospitals in India. Survey questionnaires were distributed electronically to 350 participants through Research Electronic Data Capture (REDCap, Vanderbilt University, Nashville, Tennessee, United States), with integrated written informed consent included in the survey link. Data were analyzed using descriptive statistics and represented in N (%) format.

Results: A total of 157 (44.9%) responses were obtained. Among the respondents, 67 (42.7%) were women. Participants were distributed across India, with 88 (56.1%) from southern India, 45 (28.7%) from northern India, and 23 (14.6%) from northeast India. A majority of respondents (90, 57.7%) were unaware of their institutional parental leave policies. Additionally, many respondents were unsure whether their institutions had written maternity leave policies (102, 65.0%) or paternity leave policies (100, 63.7%). Among residents, 38 (28.6%) reported having children younger than 18 years, and 8 (6.0%) were expecting children during residency training. Perceived support for residents taking parental leave was low, with 12 (46.1%) residents reporting only slight or no support from co-residents and 12 (46.1%) residents reporting only slight or no support from faculty members. Most respondents believed that the ideal time for female residents to have children was after residency (124, 79.5%). Among faculty respondents, 17 (70.8%) stated that other residents typically cover duties for residents on leave, and 10 (41.7%) believed that residents taking parental leave should extend their training period.

Conclusion: Factors that prevent a resident from having children during residency are associated with a lack of leave policies and a lack of support from co-residents and faculty members. Our data suggest that normalizing parental leave and improving policies are essential interventions to support residents with children.

## Introduction

In India, medicine is considered a highly respected and desirable profession because of its social status, career stability, and earning potential. Indian medical graduates typically complete their undergraduate medical education between 23 and 24 years of age, while postgraduate specialty training is usually completed between 27 and 29 years of age [1]. On average, physicians spend approximately three years in postgraduate education, followed by several years of registrar training, before becoming eligible for faculty positions at 29-30 years of age [2]. These years frequently coincide with important personal milestones such as marriage, childbearing, and child rearing. Consequently, the demanding years of residency training often overlap with equally significant phases in personal and family life [2].

Historically, the medical profession in India has been male-dominated. However, over the past decade, the number of women entering the medical profession has increased significantly, with recent data showing that female medical graduates now outnumber males at several institutions across the country [3-6]. Women currently constitute nearly 51% of students entering medical colleges in India [4]. Despite this progress, many women do not continue long-term clinical careers due to challenges with balancing professional and personal responsibilities. During Emergency Medicine training, long working hours, physically demanding schedules, night shifts, and the lack of flexible work environments contribute significantly to difficulties in maintaining work-life balance [3-5]. As a result, some women prefer careers in preclinical and paraclinical specialties such as anatomy, physiology, biochemistry, pathology, and microbiology, which may offer more predictable schedules and fewer clinical demands [4].

Traditionally, fathers in Indian society were considered the primary financial providers, while childcare was predominantly the responsibility of mothers. However, social attitudes regarding parenting roles have evolved considerably in recent years. Contemporary studies indicate that fathers are increasingly participating in childcare activities and valuing active involvement in parenting [4-9]. For example, previous studies have demonstrated a substantial increase in paternal participation in infant care activities compared to previous generations [6-9]. This changing social dynamic has highlighted the growing importance of parental leave policies for both mothers and fathers within medical training programs.

To the best of our knowledge, limited research has been conducted in India regarding the perceptions of Emergency Medicine (EM) residents and faculty toward parental leave and its impact on training, workload distribution, and workplace support. Understanding these perceptions is important to guide institutional policies that support resident well-being and promote a balanced training environment. Therefore, the present study aimed to analyze the perceptions of Indian Emergency Medicine residents and faculty regarding parental leave during residency training.

## Materials and methods

A multicenter prospective observational study was conducted between September 2022 and January 2023 among EM residents and faculty members affiliated with George Washington University-supported three-year emergency medicine training programs in India. The study was carried out across tertiary care teaching hospitals participating in the academic collaboration program. A total of 350 participants, which included 300 residents and 50 faculty members from six Master of Emergency Medicine (MEM) sites. Those enrolled were invited to participate in an anonymous, electronically distributed survey via the secure web-based software, Research Electronic Data Capture (REDCap, Vanderbilt University, Nashville, Tennessee, United States). Residents of all three academic years were included along with faculty who were actively affiliated with the program irrespective of marital or childbearing status. Former residents and faculty no longer working with the MEM program were excluded from the study. The survey link included integrated written informed consent, and participation was voluntary. Reminder emails were sent periodically to non-respondents to improve the response rate.

A structured 41-item anonymous questionnaire was adapted from previous survey instruments assessing perceptions regarding parental leave among medical trainees and faculty members [8,9]. Minor modifications were made to suit the Indian emergency medicine residency training context. The questionnaire collected demographic information, including age, gender, relationship status, employment status of the respondent and spouse/partner, and parental status. Additional questions assessed parental leave taken during residency, its duration, perceived support from co-residents and faculty members, opinions regarding parental leave, future plans for having children during residency, and barriers to taking parental leave. Respondents were also asked about their awareness and knowledge of existing institutional maternity and paternity leave policies. The questionnaire required approximately 10 minutes to complete. Anonymous responses were collected, and reminder emails were sent to non-respondents up to five times.

Data were collected and managed using REDCap electronic data capture tools. Statistical analysis was performed using Statistical Package for the Social Sciences (SPSS) software version 26.0 (IBM, Armonk, New York, United States). Descriptive statistics were presented in N (%) format with percentages rounded to one decimal place. Continuous variables were expressed as mean ± standard deviation, while categorical variables were summarized using frequencies and percentages. The Chi-square test and Fisher’s exact test were used to evaluate associations between principal variables of interest and dependent variables. A p-value of <0.05 was considered statistically significant. The study was approved by the Meenakshi Mission Hospital and Research Centre Institutional Review Board, Madurai, India (approval number: IORG0007720, date: December 13, 2022). Written informed consent was obtained electronically from all participants prior to survey participation.

## Results

A total of 157 (44.9%) responses were received during the study period, including 133 (84.7%) Emergency Medicine (EM) residents and 24 (15.3%) EM faculty members. Among the resident respondents, 50 (37.9%) were in their first year of residency, 47 (35.6%) were in their second year, and 35 (26.5%) were in their final year of residency training. The mean age of resident participants was 29 years. The demographic characteristics of the study participants are presented in Table 1.

**Table 1:** Demographic characteristics of participants by qualification (N = 157) S: Statistically Significant, NS: Not Statistically Significant, EM: Emergency Medicine.

		EM Postgraduates (n=133)	EM Faculty (n=24)	Total (N=157)	p-value Significance
Gender	Male	75 (56.4%)	15 (62.5%)	90 (57.3%)	0.658 (NS)
	Female	58 (43.6%)	9 (37.5%)	67 (42.7%)	
Age group	18-25 years	3 (2.3%)	0 (0.0%)	3 (1.9%)	0.016 (S)
	26-40 years	126 (94.7%)	20 (83.3%)	146 (93.0%)	
	41-60 years	4 (3.0%)	4 (16.7%)	8 (5.1%)	
Relationship status	Married	72 (54.1%)	22 (91.7%)	94 (59.9%)	0.003 (S)
	Single	60 (45.1%)	2 (8.3%)	62 (39.5%)	
	Divorced/Widowed/Separated	1 (0.8%)	0 (0.0%)	1 (0.6%)	
Employment status	Full-time (>40 hrs)	124 (93.2%)	24 (100%)	148 (94.3%)	0.423 (NS)
	Part-time	6 (4.5%)	0 (0.0%)	6 (3.8%)	
	On leave	3 (2.3%)	0 (0.0%)	3 (1.9%)	
Spouse/partner profession	Non-physician	26 (36.1%)	10 (45.5%)	36 (38.3%)	0.159 (NS)
	Physician	25 (34.7%)	10 (45.5%)	35 (37.2%)	
	Resident	21 (29.2%)	2 (9.1%)	23 (24.5%)	
Perceived support during parental leave among residents	Not at all	2 (18.2%)	1 (6.7%)	3 (11.5%)	0.063 (NS)
	Slightly	3 (27.3%)	6 (40.0%)	9 (34.6%)	
	Somewhat	0 (0.0%)	5 (33.3%)	5 (19.2%)	
	Moderately	3 (27.3%)	0 (0.0%)	3 (11.5%)	
	Extremely	3 (27.3%)	3 (20.0%)	6 (23.1%)	
Perceived faculty support during parental leave	Not at all	1 (9.1%)	4 (26.7%)	5 (19.2%)	0.764 (NS)
	Slightly	3 (27.3%)	4 (26.7%)	7 (26.9%)	
	Somewhat	3 (27.3%)	4 (26.7%)	7 (26.9%)	
	Moderately	2 (18.2%)	1 (6.7%)	3 (11.5%)	
	Extremely	2 (18.2%)	2 (13.3%)	4 (15.4%)	
Co-resident parental leave and related perceptions	Yes	35 (46.7%)	32 (55.2%)	67 (50.4%)	0.331 (NS)
	No	40 (53.3%)	26 (44.8%)	66 (49.6%)	

Demographics and relationship status

Among resident respondents, 72 (54.1%) were married or in a domestic partnership. Faculty members were significantly more likely to be married compared to residents (22 (91.7%) vs. 72 (54.1%); p=0.003). The majority of participants (148, 94.3%) reported working full-time (>40 hours per week).

Knowledge of parental leave policies

Only 66 (42.3%) respondents reported awareness of their institution’s current parental leave policies. Awareness of a written maternity leave policy was reported by 26 (16.6%) respondents, while awareness of a written paternity leave policy was reported by 11 (7.0%) respondents. Faculty members were significantly more likely than residents to report awareness of written maternity leave policies (9 (37.5%) vs. 17 (12.8%); p=0.01) and paternity leave policies (6 (25.0%) vs. 5 (3.8%); p=0.003) (Table 2).

**Table 2:** Questions for both residents and faculty of the training programs S: Statistically Significant, NS: Not Statistically Significant, EM: Emergency Medicine.

Variable	Response	EM Postgraduates (n=133)	EM Faculty (n=24)	Total (N=157)	p-value
Awareness of current parental leave policy	Yes	52 (39.4%)	14 (58.3%)	66 (42.3%)	0.084 (NS)
	No	80 (60.6%)	10 (41.7%)	90 (57.7%)	
Written maternity leave policy exists	Yes	17 (12.8%)	9 (37.5%)	26 (16.6%)	0.010 (S)
	No	25 (18.8%)	4 (16.7%)	29 (18.5%)	
	Unsure	91 (68.4%)	11 (45.8%)	102 (65.0%)	
Duration of maternity leave (if available)	2-10 weeks	4 (36.4%)	1 (14.3%)	5 (27.8%)	0.256 (NS)
	10-20 weeks	4 (36.4%)	1 (14.3%)	5 (27.8%)	
	20-24 weeks	3 (27.3%)	4 (57.1%)	7 (38.9%)	
	Unanswered	0 (0.0%)	1 (14.3%)	1 (5.6%)	
Written paternity leave policy exists	Yes	5 (3.8%)	6 (25.0%)	11 (7.0%)	0.003 (S)
	No	39 (29.3%)	7 (29.2%)	46 (29.3%)	
	Unsure	89 (66.9%)	11 (45.8%)	100 (63.7%)	
Duration of parental leave (if available)	<2 weeks	2 (50.0%)	2 (66.7%)	4 (57.1%)	0.646 (NS)
	2-4 weeks	1 (25.0%)	1 (33.3%)	2 (28.6%)	
	4-6 weeks	1 (25.0%)	0 (0.0%)	1 (14.3%)	
Best time for female residents to have children	Before residency	15 (11.4%)	4 (16.7%)	19 (12.2%)	0.763 (NS)
	During residency	11 (8.3%)	2 (8.3%)	13 (8.3%)	
	After residency	106 (80.3%)	18 (75.0%)	124 (79.5%)	
Best time for male residents to have children	Before residency	7 (5.3%)	4 (16.7%)	11 (7.1%)	0.130 (NS)
	During residency	23 (17.6%)	3 (12.5%)	26 (16.8%)	
	After residency	101 (77.1%)	17 (70.8%)	118 (76.1%)	
Maternity leave causes strain	Yes	71 (53.8%)	17 (70.8%)	88 (56.4%)	0.121 (NS)
	No	61 (46.2%)	7 (29.2%)	68 (43.6%)	
Paternity leave causes strain	Yes	72 (55.0%)	18 (75.0%)	90 (58.1%)	0.067 (NS)
	No	59 (45.0%)	6 (25.0%)	65 (41.9%)	
Recommended maternity leave duration	None	2 (1.5%)	0 (0.0%)	2 (1.3%)	0.302 (NS)
	2-4 weeks	3 (2.3%)	0 (0.0%)	3 (1.9%)	
	4-5 weeks	12 (9.0%)	0 (0.0%)	12 (7.6%)	
	>6 weeks	102 (76.7%)	23 (95.8%)	125 (79.6%)	
	Unsure	14 (10.5%)	1 (4.2%)	15 (9.6%)	
Recommended paternal leave duration	None	2 (1.5%)	0 (0.0%)	2 (1.3%)	0.160 (NS)
	<2 weeks	24 (18.0%)	5 (20.8%)	29 (18.5%)	
	2-4 weeks	36 (27.1%)	12 (50.0%)	48 (30.6%)	
	4-5 weeks	25 (18.8%)	4 (16.7%)	29 (18.5%)	
	>6 weeks	29 (21.8%)	3 (12.5%)	32 (20.4%)	
	Unsure	17 (12.8%)	0 (0.0%)	17 (10.8%)	

Residents with children

Among the residents surveyed, 38 (28.6%) reported having children younger than 18 years. Of these, 25 (18.8%) reported having children during their residency years, while 8 (21.1%) were currently expecting children during residency. Although a greater proportion of female residents were married, more male residents reported plans to have children during residency compared to female residents (18 (24.0%) vs. 11 (19.0%)).

Parental leave utilization

Only 25 (18.8%) residents reported taking or planning to take parental leave during residency. Among these residents, 16 (61.5%) reported taking more than four weeks of leave. Female residents were significantly more likely than male residents to take more than six weeks of leave (8 (53.3%) vs. 1 (9.1%); p<0.01). Female residents also demonstrated greater awareness regarding parental leave policies compared to male residents, including awareness of general parental leave policies (42 (56.0%) vs. 33 (44.0%)), maternity leave policies (55 (59.1%) vs. 38 (40.9%)), and paternity leave policies (52 (57.1%) vs. 39 (42.9%): p=0.004).

Perceived support and financial impact

Among the 38 (28.6%) residents who reported being parents, 22 (57.9%) reported loss of pay when taking time off to care for a sick child during residency. Among residents who took formal parental leave, only 9 (34.6%) reported feeling moderately to extremely supported by co-residents, including 3 (11.5%) who felt moderately supported and 6 (23.1%) who felt extremely supported. Similarly, only 7 (26.9%) residents reported feeling moderately to extremely supported by faculty members during parental leave, including 3 (11.5%) who felt moderately supported and 4 (15.4%) who felt extremely supported (Table 3).

**Table 3:** Responses of MEM residents regarding parental leave MEM: Master of Emergency Medicine, S: Statistically Significant, NS: Not Statistically Significant.

Responses of MEM Residents Regarding Parental Leave		Male (n=75)	Female (n=58)	Total (N=133)	p-value Significance
Year of residency	1st year	32 (42.7%)	18 (31.6%)	50 (37.9%)	0.424 (NS)
	2nd year	25 (33.3%)	22 (38.6%)	47 (35.6%)	
	3rd year	18 (24.0%)	17 (29.8%)	35 (26.5%)	
Had children during residency	Yes	13 (17.3%)	12 (20.7%)	25 (18.8%)	0.847 (NS)
	No	57 (76.0%)	43 (74.1%)	100 (75.2%)	
Currently expecting		5 (6.7%)	3 (5.2%)	8 (6.0%)	
Consider future children	Yes	18 (24.0%)	11 (19.0%)	29 (21.8%)	0.486 (NS)
	No	57 (76.0%)	47 (81.0%)	104 (78.2%)	
Parent/guardian of child <18	Yes	17 (22.7%)	21 (36.2%)	38 (28.6%)	0.087 (NS)
	No	58 (77.3%)	37 (63.8%)	95 (71.4%)	
You are usually the one to care for the child		4 (23.5%)	4 (19.0%)	8 (21.1%)	0.483 (NS)
Spouse/partner/co-parent usually cares for the child		9 (52.9%)	7 (33.3%)	16 (42.1%)	
Shares responsibility equally		3 (17.6%)	7 (33.3%)	10 (26.3%)	
Call someone else to care for the child		1 (5.9%)	3 (14.3%)	4 (10.5%)	
Lose pay for sick child leave	Yes	9 (52.9%)	13 (61.9%)	22 (57.9%)	0.578 (NS)
	No	8 (47.1%)	8 (38.1%)	16 (42.1%)	
Took/planning parental leave	Yes	11 (14.7%)	14 (24.1%)	25 (18.8%)	0.166 (NS)
	No	64 (85.3%)	44 (75.9%)	108 (81.2%)	
Duration of parental leave	<2 weeks	1 (9.1%)	5 (33.3%)	6 (23.1%)	0.004 (S)
	2-4 weeks	4 (36.4%)	0 (0.0%)	4 (15.4%)	
	4-6 weeks	5 (45.5%)	2 (13.3%)	7 (26.9%)	
	>6 weeks	1 (9.1%)	8 (53.3%)	9 (34.6%)	

Most residents believed that the ideal time for female residents to have children was after residency training, as reported by 124 (79.5%) respondents. Similarly, 118 (76.1%) respondents believed that the ideal time for male residents to have children was after residency training. More than half of residents without children believed that maternity and paternity leave placed an unreasonable strain on other residents in the program, with 50 (53.2%) and 50 (53.8%) respondents reporting this perception, respectively. Among faculty respondents, 17 (70.8%) reported that other residents usually covered duties for residents taking parental leave, while 10 (41.7%) reported that residents taking parental leave were required to extend their training (Table 4).

**Table 4:** Responses of residents with and without children S: Statistically Significant, NS: Not Statistically Significant.

Variable		Residents With Children (n=38)	Residents Without Children (n=94)	Total (N=132)	p-value Significance
Maternity leave causes strain on residents	Yes	21 (55.3%)	50 (53.2%)	71 (53.8%)	0.829 (NS)
	No	17 (44.7%)	44 (46.8%)	61 (46.2%)	
Paternity leave causes strain on residents	Yes	22 (57.9%)	50 (53.8%)	72 (55.0%)	0.666 (NS)
	No	16 (42.1%)	43 (46.2%)	59 (45.0%)	
Best time for male residents to have children	Before residency	4 (10.5%)	3 (3.2%)	7 (5.3%)	0.238 (NS)
	During residency	6 (15.8%)	17 (18.3%)	23 (17.6%)	
	After residency	28 (73.7%)	73 (78.5%)	101 (77.1%)	
Best time for female residents to have children	Before residency	8 (21.1%)	7 (7.4%)	15 (17.4%)	0.037 (S)
	During residency	1 (2.6%)	10 (10.6%)	11 (8.3%)	
	After residency	29 (76.3%)	77 (81.9%)	106 (80.3%)	

Faculty opinions regarding parental leave

Among faculty respondents, most believed that the ideal time for residents to have children was after completion of residency training, including 18 (75.0%) for female residents and 17 (70.8%) for male residents. Additionally, 10 (41.7%) faculty members reported that residents who took parental leave were required to extend their residency training period (Table 5).

**Table 5:** Survey questions to the emergency medicine faculty (N=24) NS: Not Statistically Significant.

Variable		Male (n=15)	Female (n=9)	Total (N=24)	p-value (Sig)
Who covers residents on parental leave?	Other residents	9 (60.0%)	8 (88.9%)	17 (70.8%)	0.284 (NS)
	Attending emergency physicians	4 (26.7%)	1 (11.1%)	5 (20.8%)	
	Coverage not necessary	2 (13.3%)	0 (0.0%)	2 (8.3%)	
Extension of residency after parental leave	Yes	8 (53.3%)	2 (22.2%)	10 (41.7%)	0.292 (NS)
	Depends on competency	3 (20.0%)	2 (22.2%)	5 (20.8%)	
	Depends on the duration of leave	3 (20.0%)	2 (22.2%)	5 (20.8%)	
	Unsure	1 (6.7%)	3 (33.3%)	4 (16.7%)	
Residents extending training due to parental leave (last 3 years)	None	7 (46.7%)	5 (55.6%)	12 (50.0%)	0.404 (NS)
	1 resident	1 (6.7%)	1 (11.1%)	2 (8.3%)	
	≥3 residents	6 (40.0%)	1 (11.1%)	7 (29.2%)	
	Unsure	1 (6.7%)	2 (22.2%)	3 (12.5%)	

## Discussion

In this multicenter study, a substantial proportion of respondents were unaware of the parental leave policies at their respective institutions. Most residents believed that the ideal time to have children was after completing residency training, and the lack of clear parental leave policies was identified as a major barrier to parenthood during residency. These findings highlight the need for greater institutional awareness and standardized parental leave guidelines within Emergency Medicine training programs in India.

Limited awareness of parental leave policies may contribute to stigma around its use during residency training. Among residents who reported taking parental leave, many perceived inadequate support from co-residents and faculty members. This perception may be related to concerns regarding the redistribution of clinical workload and increased responsibilities for other residents during periods of leave. Additionally, several respondents reported that residents on parental leave were often required to extend their training.

Parental leave policies, family support, and workplace systems can significantly influence resident well-being and professional satisfaction [8,10-12]. Previous studies conducted in the United States have demonstrated that family-related stress and inadequate work-life balance contribute substantially to resident burnout and depression [13,14]. Existing parental leave policies are frequently perceived as insufficient, particularly regarding the duration of leave permitted for resident physicians. A Canadian survey similarly found that many trainees were unaware of institutional parental leave policies, yet emphasized the importance of formal maternity and paternity leave policies within residency programs [15]. The same study also found that some residents perceived pregnancy among peers as negatively affecting workload distribution and increasing on-call responsibilities [15]. In the present study, 18 (75.0%) faculty members and 72 (55.0%) residents believed that parental leave imposed an unreasonable burden on other residents within the training program. Overall perceptions toward maternity and paternity leave were predominantly unfavorable, suggesting the persistence of institutional and cultural challenges surrounding parenthood during residency training.

Although residency training commonly coincides with the typical childbearing years, most residents preferred to postpone parenthood until after completing training. This finding reflects the perceived inadequacy of institutional support systems available for resident parents. Importantly, these concerns are not limited to female residents alone. Increasing paternal involvement in childcare and parenting responsibilities among younger generations further emphasizes the importance of equitable parental leave policies for both mothers and fathers within residency programs. Introduction of creche/daycare facilities, protocolized durations of leave, adjustable working hours, and facilitative working environment are areas to consider for improvements and potential future directions.

Limitations

This study has several limitations. As the survey was conducted among a subset of Emergency Medicine residency programs in India, the findings may not be generalizable to all residency programs nationally or internationally. The response rate was 44.9%, despite repeated reminders via social media and email, which may have introduced response bias. Based on the study design, the potential for selection bias, nonresponse bias, and reliance on self-reported perceptions are considered potential limitations. Additionally, first-year residents constituted a larger proportion of respondents and may therefore be overrepresented in the study findings. The number of faculty participants was also limited. Future multicenter and multinational studies involving larger, more diverse participant populations are needed to further evaluate perceptions of parental leave policies and to identify best practices for implementation across residency training programs.

## Conclusions

This multicenter study highlights significant gaps in awareness, availability, and perception of parental leave policies among Emergency Medicine residents and faculty members in India. Lack of standardized parental leave policies, inadequate institutional support, and perceived stigma surrounding pregnancy and parenthood during residency were identified as important barriers influencing residents’ decisions regarding parenthood during training. The findings emphasize the need for formal, transparent, and universally applicable parental leave policies within residency programs. Improved communication regarding existing leave policies, greater scheduling flexibility, and supportive workplace environments may help reduce the challenges faced by resident parents. The development of mentorship and support systems for residents with children may further promote residents' well-being and work-life balance. As one of the first multicenter studies in India to evaluate perceptions of parental leave in Emergency Medicine training programs, this study highlights the importance of creating equitable, family-friendly residency environments that support both maternal and paternal responsibilities during medical training.
